# Assessing the state of Web-based communication for public health: a systematic review

**DOI:** 10.1093/eurpub/ckac130.056

**Published:** 2022-10-25

**Authors:** E Ceretti, L Covolo, F Cappellini, A Nanni, S Sorosina, M Taranto, A Gasparini, P De Castro, S Brusaferro, U Gelatti

**Affiliations:** 1 Department of Medicine, Università degli Studi di Brescia, Brescia, Italy; 2 Post-graduate School of Public Health, Università degli Studi di Brescia, Brescia, Italy; 3 Italian National Institute of Health, Rome, Italy

## Abstract

**Background:**

Communicating strategically is a key issue for health organisations and, over the past decade, healthcare communication via social media and websites has generated a great deal of studies. As for systematic reviews, there is, however, fragmentary evidence on this type of communication. The aim of this research was to summarise the evidence on Web institutional health communication for public health authorities to evaluate aim-specific key points based on existing studies.

**Methods:**

Guided by the PRISMA statement, we conducted a comprehensive review across two electronic databases (PubMed and Web of Science) from 2011 until 7 October 2021, searching for studies investigating institutional health communication. Two independent researchers reviewed the articles for inclusion, and assessment of methodological quality was based on the Kmet appraisal checklist.

**Results:**

78 articles were selected. Most of the studies targeted health promotion/disease prevention (n = 35), followed by crisis communication (n = 24), general health (n = 13), and misinformation correction/health promotion (n = 6). Engagement and message framing were the most analysed aspects. Few studies focused on campaign effectiveness. Only 18 studies had an experimental design. Kmet evaluation was used to distinguish studies presenting a solid structure from lacking studies. In particular, considering the 0·75-point threshold, out of 74 studies, 28 were excluded (37·8% of the total). Studies above this threshold were used to identify a series of aim-specific and medium-specific suggestions, as communication strategies employed differ quite greatly.

**Conclusions:**

Overall, findings suggest that no single strategy works best in the case of Web-based healthcare communication. The extreme variability of outcomes and the lack of a unitary measure for assessing the end-points of a specific campaign or study leads us to reconsider the tools we use to evaluate the efficacy of Web-based health communication.

**Key messages:**

- This systematic review provides an overview on Web-based health communication. Results suggest that no single strategy works best and the need to reassess its evaluation tools.
- Communicating strategically is key for health organisations. This systematic review analyses a corpus of texts, in an effort to summarise and analyse existing, albeit fragmentary, evidence.

